# 4-phenylbutyric acid improves sepsis-induced cardiac dysfunction by modulating amino acid metabolism and lipid metabolism via Comt/Ptgs2/Ppara

**DOI:** 10.1007/s11306-024-02112-3

**Published:** 2024-04-19

**Authors:** Yuanqun Zhou, Yu Zhu, Yue Wu, Xinming Xiang, Xingnan Ouyang, Liangming Liu, Tao Li

**Affiliations:** State Key Laboratory of Trauma, Burns and Combined Injury, Shock and Transfusion of Research Institute of Surgery, Daping Hospital, Army Medical University, Chongqing, China

**Keywords:** 4-phenylbutyric acid, Cardiac dysfunction, Sepsis, Metabolism

## Abstract

**Introduction:**

Cardiac dysfunction after sepsis the most common and severe sepsis-related organ failure. The severity of cardiac damage in sepsis patients was positively associated to mortality. It is important to look for drugs targeting sepsis-induced cardiac damage. Our previous studies found that 4-phenylbutyric acid (PBA) was beneficial to septic shock by improving cardiovascular function and survival, while the specific mechanism is unclear.

**Objectives:**

We aimed to explore the specific mechanism and PBA for protecting cardiac function in sepsis.

**Methods:**

The cecal ligation and puncture-induced septic shock models were used to observe the therapeutic effects of PBA on myocardial contractility and the serum levels of cardiac troponin-T. The mechanisms of PBA against sepsis were explored by metabolomics and network pharmacology.

**Results:**

The results showed that PBA alleviated the sepsis-induced cardiac damage. The metabolomics results showed that there were 28 metabolites involving in the therapeutic effects of PBA against sepsis. According to network pharmacology, 11 hub genes were found that were involved in lipid metabolism and amino acid transport following PBA treatment. The further integrated analysis focused on 7 key targets, including Comt, Slc6a4, Maoa, Ppara, Pparg, Ptgs2 and Trpv1, as well as their core metabolites and pathways. In an in vitro assay, PBA effectively inhibited sepsis-induced reductions in Comt, Ptgs2 and Ppara after sepsis.

**Conclusions:**

PBA protects sepsis-induced cardiac injury by targeting Comt/Ptgs2/Ppara, which regulates amino acid metabolism and lipid metabolism. The study reveals the complicated mechanisms of PBA against sepsis.

**Supplementary Information:**

The online version contains supplementary material available at 10.1007/s11306-024-02112-3.

## Introduction

Sepsis is a common complication of severe burns, shock, and infection (Singer et al., 2016), as well as in surgical patients. The sepsis related multiple organ dysfunction syndromes was a leading cause of death in patients admitted to intensive care units. As a vital organ, the heart was most susceptible to sepsis-induced organ damage, which might lead to poor prognosis (Hollenberg & Singer, 2021). Some researchers demonstrated that myocardial dysfunction was an independent risk factor for death in individuals following sepsis and occurred early in the development of the disease (Carbone et al., 2022). The severity of heart injury has been demonstrated to aggravate sepsis-induced death. Therefore, it is of great significance to find effective measures to protect myocardial function to improve the outcome of sepsis.

A resent research considered that the mechanisms of cardiac dysfunction following sepsis involved in inflammatory mediator dysregulation, mitochondrial dysfunction, oxidative stress, calcium regulation disorder, microcirculatory injury and so on (Zhang & Ning, 2021). There was a lack of targeting treatment measures in clinical practice according to above mechanisms. Previous studies found the usage of single antioxidant stress or anti-inflammatory drugs only improved cardiac function slightly in sepsis, the therapeutic effect was limited (Hanna et al., 2022). Our previous study found that 4-phenylbutyric acid (PBA) improved survival in rats after sepsis by improving cardiovascular function, the blood flow of vital organs as well as the delivery and utilization of oxygen were improved (Kuang et al., 2021), which indicated that PBA may be a prospective therapy treatment to correct cardiovascular dysfunction in patients with sepsis and septic shock. Basic studies found that PBA was an ammonia scavenger and was used to correct urea cycle disorders in clinic under the trade name Buphenyl (Kolb et al., 2015). A large of evidences demonstrated that PBA was a low molecular weight chemical chaperone acting as an ER stress inhibitor which prevented misfolded protein aggregation and alleviated endoplasmic reticulum (ER) stress (Zito, 2019). Recent studies showed that PBA also acted as a histone deacetylase inhibitor, it could activate a gene transcription program and then modulate proteostasis (Li et al., 2021). In addition, some studies suggested that PBA could regulate mitochondrial function by increasing β-oxidation and participate in mitochondrial biogenesis (Tiwari et al., 2022). A recent study showed that PBA was an mRNA translation attenuator, reducing translation levels from bacteria to mammalian cells, both in vitro and in cells (Stein et al., 2022). These studies indicated that PBA participated in a variety of intracellular processes. However, the therapeutic role of PBA in sepsis and its molecular mechanisms need to be further investigated.

The present study aimed to explore the specific mechanism and PBA for protecting cardiac function in sepsis. The results will provide an experimental basis for the treatment of myocardial injury in sepsis, and provide novel ideas for the search for therapeutic targets for sepsis.

## Methods

### Materials, animals and model establishment

4-phenylbutyric acid (PBA, #P21005) was purchased from Sigma-Aldrich. Sprague–Dawley rats (220–240 g) were obtained from the animal care center of Daping Hospital, Army Military Medical University (license No. SCXK[Yu]20,170,002). Cecal ligation and puncture (CLP) was used to replicate the sepsis model in rats as described previously (She et al., 2021). Briefly, rats were anaesthetized with sodium pentobarbital (30 mg/kg), and then the cecum was exposed and ligated by 7.5 mm to its end. The ligated cecum was punctured (≈ 1.5 mm) with a triangular needle (sham-operated rats only received cecum ligation and no puncture). Feces were allowed to flow into the abdominal cavity. After the closure of the abdomen, rats were returned to the cages. 12 h after CLP, the cardiac tissues were taken for the following experiments. Rats in PBA group were tail-vein injected with PBA (5 mg/kg) 30 min prior to the operation. Another dose of PBA (5 mg/kg) was administered 12 h after the operation.

### Isolation of rat ventricular cardiomyocytes

The heart was removed and washed with ice cold Krebs-Henseleit Buffer (KHB) containing (in mM): 140 NaCl, 5.0 KCl, 1.0 MgCl_2_, 5 HEPES, and 10 glucose (pH 7.35). Then the heart was perfused with KHB using Langendorff apparatus, then switched to digestion buffer (KHB, 240 units/mL collagenase type II (Worthington Biochemical Corporation, NJ, USA) and bovine serum albumin (BSA)). All solutions were heated to 37 °C prior to perfusion through the heart and aerated with 95% oxygen and 5% CO_2_ to maintain pH at 7.35. The heart was continuously checked to monitor digestion and then the left ventricle was separated from the rest of the heart and minced. The cardiomyocytes were then filtered, resuspended, and equilibrated in room temperature KHB with 200 μM CaCl_2_ and 1% BSA. Extracellular Ca^2+^ was added incrementally back to 1.8 mM, and then experiments were performed after allowing cardiomyocytes to rest for at least 30 min.

### Measurement of cardiomyocytes contractility

Mechanical properties of cardiomyocytes were assessed using an IonOptix soft-edge system (IonOptix Corporation, Milton, MA, USA). Myocytes were placed in a chamber on the microscope stage and superfused with KHB with 1.8 mM CaCl_2_. The cells were field stimulated with suprathreshold voltage at 1 Hz. Once the steady state was reached, a minimum of 15 contractions was recorded. IonWIizard software was used to measure the percentage of the systolic amplitude (SS), time to peak shortening, time to 50% baseline.

### Metabolomics profiling

Metabolomics profiling was performed in tissues of septic rats and sham control by using a UHPLC system (Vanquish, Thermo Fisher Scientific) with a UPLC BEH Amide column (2.1 mm × 100 mm,1.7 μm) coupled to a Q-Exactive HFX mass spectrometer (Orbitrap MS, Thermo, United States). The QE HFX mass spectrometer was used to acquire MS/MS spectra on information-dependent acquisition (IDA) mode in the control of the acquisition software (Xcalibur, Thermo, United States). The resulting three-dimensional data involving the peak number, sample name, and normalized peak area were inputted into SIMCA-P software version 13.0 (Umetrics AB, Umea, Sweden) for multivariate statistical analysis. Principal component analysis (PCA), partial least squares discriminant analysis (PLS-DA), model validation diagram, orthogonal partial least squares discriminant analysis (OPLS-DA) and (V + S)-plot scatter diagram was performed, respectively. The differential metabolites of PBA and sepsis were identified by screening the RT of T test *p*-value < 0.05, pcorr-absolute value < 0.5 and VIP-value > 1.0, and then combining with the human metabolomic database (HMDB) (https://hmdb.ca/), that is, the differential metabolites of PBA and sepsis were obtained. The HMDB database and related literatures were searched with accurate quality to identify the differential metabolites. The compounds were identified within 5 ppm mass accuracy.

### Metabolic pathway analysis, heatmaps analysis and the metabolite-gene network construction

The genes were imported into the MetaboAnalyst 5.0 database (https://www.metaboanalyst.ca/faces/home.xhtml) for heatmaps analysis and metabolic pathway analysis, and then the differential metabolites were imported into Metscape for the metabolite-gene network construction. Then, the related genes of the metabolites, a compounds-candidate targets-metabolite related gene-metabolites-metabolic pathways-disease network construction was constructed.

### Network pharmacology construction

The molecular targets of PBA were collected from Swiss Target database (http://www.swisstargetprediction.ch/) and Similarity ensemble approach (http://sea.bkslab.org/). Sepsis-related targets were obtained from Genecards (https://www.genecards.org/) and DisGeNET (http://www.disgenet.org/). The intersection of PBA-related genes and sepsis-related targets was considered the predicted target of PBA on sepsis.

These targets were imported into UniProtKB (http://www.uniprot.org/) to standardize the gene and protein names. Metascape (http://metascape.org/gp/index.html) was employed for integrated functional enrichment, gene annotation, and interactive group analysis. The cellular components, molecular function, biological process, and biological pathway were then analyzed by using FunRich platform. *p* < 0.05 was considered statistically significant. A protein–protein interaction (PPI) network was established by STRING 11.5 (https://string-db.org/) and Cytoscape 3.9.0. Hub genes were obtained using CytoHubba in Cytoscape. GeneMANIA (http://www.genemania.org) was applied to construct a gene–gene interaction network for hub genes.

### Microarray data

To explore the prognostic signature of hub genes, the microarray data for sepsis were downloaded from the Gene Expression Omnibus (GEO) database (https://www.ncbi.nlm.nih.gov/geo/) under accession numbers GSE79962. Background correction and quantile normalization were performed using LIMMA software package. Based on the gene expression matrix, CIBERSORT software was used to characterize the relative proportion and corresponding *p*-value of 22 immune cells in each sample in the dataset GSE79962.

### Statistical analysis

The data were represented as means ± SD. Statistical analysis was performed by one-way ANOVA using GraphPad Prism 9 (GraphPad Software, Inc., La Jolla, CA, USA). A *p*-value of < 0.05 was considered to be statistically significant.

## Results

### The protective effect of PBA on myocardium in septic rats

**Fig. 1:**
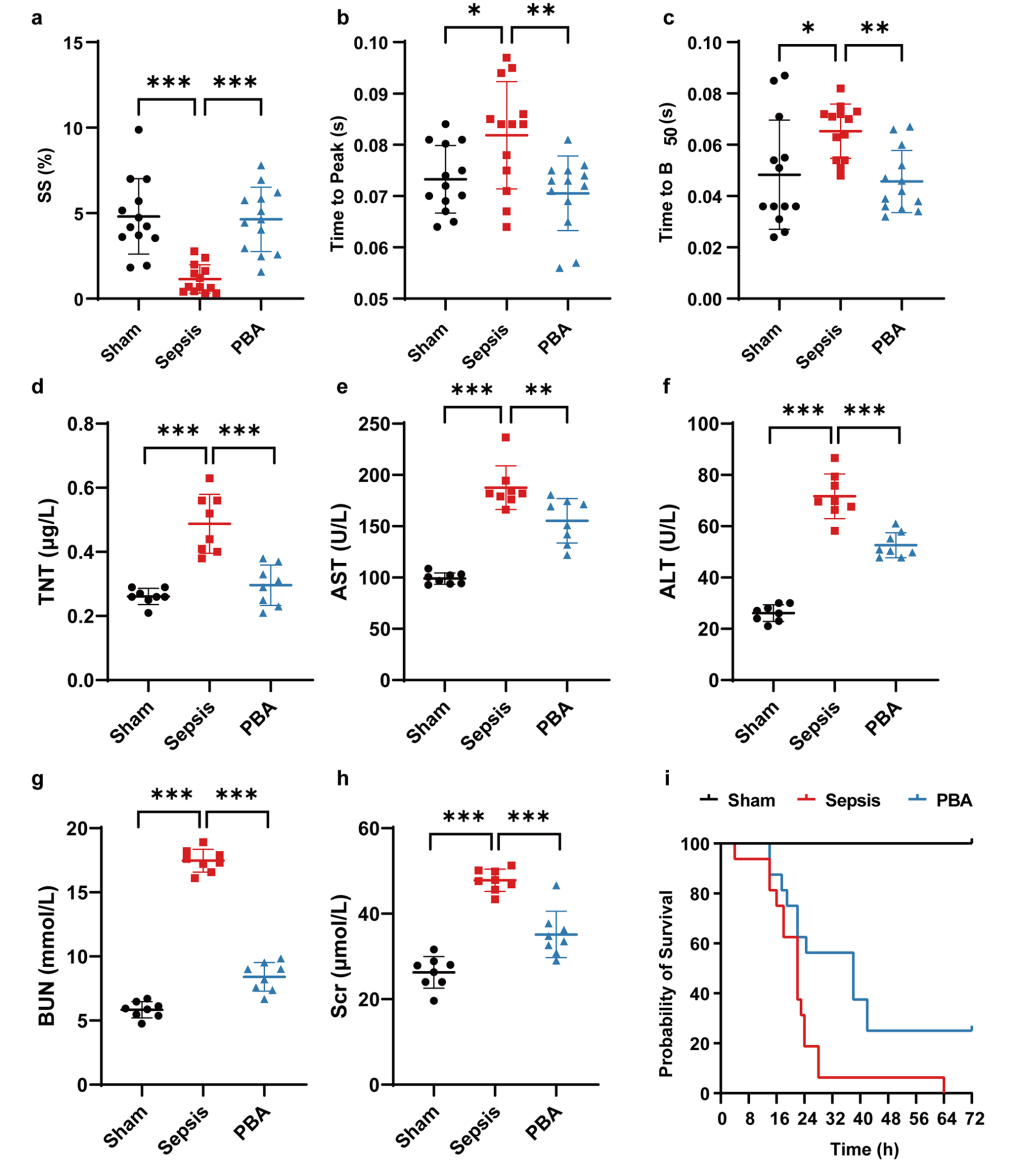
Effects of PBA on cardiac function in septic rats. (**a**) The maximum percentage of cell contraction. *n* = 13. (**b**) The time when cell contraction reached the peak. *n* = 13. (**c**) The time to 50% relaxation. *n* = 13. (**d**-**h**) Vital organs function. TNT, Troponin T. AST, Aspartate transaminase. ALT, Alanine aminotransferase. Scr, serum creatinine. BUN, blood urea nitrogen. *n* = 8. (**i**), Survival rate. *n* = 16. Results are represented as mean ± SD. *, *p*<0.05; **, *p*<0.01; ***, *p*<0.001

To observe the protective effects of PBA on cardiac function for sepsis, PBA (20 mg/kg) was injected into rats via tail vein immediately and 12 h after sepsis. The contractile and diastolic properties of cardiomyocytes was measured. With 1 Hz stimulation, the contractility of cardiomyocytes in the septic rats was significantly suppressed, as the amplitude of contraction (SS%) was significantly reduced by more than 50%, and the time to peak and 50% relaxation were prolonged (Fig. 1a-c). PBA administration could improve the contractility of cardiomyocytes in sepsis. The amplitude of contraction (SS%) was significantly higher in PBA group than that in sepsis group (PBA:4.64 ± 1.88% vs. Sepsis:1.15 ± 0.83%), and the time to peak (PBA: 0.071 ± 0.007s vs. Sepsis: 0.08 ± 0.01s) and 50% relaxation (PBA :0.045 ± 0.012s vs. Sepsis: 0.06 ± 0.01s) were reduced in the PBA group. The results indicate that PBA induce a direct positive inotropic effect on cardiomyocytes in sepsis, and improve the contractile function by increasing the contraction/diastole rate. The further study found that TnT, a biomarker of cardiac injury, was significantly higher in the septic rats as compared with control rats, while the TnT levels was reduced following PBA treatment (Fig. 1d). At the same time, PBA treatment could significantly reduce the liver and kidney function damage in sepsis (Fig. 1e-h). Survival results showed that the survival rate of rats at 72 h was significantly improved after PBA treatment as compared with sepsis group (Fig. 1i).

### Effect of PBA on cardiac metabolism in septic rats

Previous studies focused on effects of PBA as an ERS inhibitor, while PBA is a useful small molecule which has shown multiple biological functions. To explore the mechanism of myocardial protective effect of PBA on sepsis, untargeted metabolomics was applied to detect the changes of cardiac metabolites after PBA treatment. The metabolic profile was obtained in both positive and negative ion modes by UPLC-MS. In the two modes, 7174 ion peaks were collected by XCMS software for subsequent multivariate analysis. PLS-DA was carried out to district the metabolic profiles of the experimental groups (Fig S1a). Permutation test with 200 permutations showed that Q^2^ and R^2^ value were lower than the original points, which suggested that the established model was deemed to be successful (Fig S1b and c). As a result, the metabolic profile of the three tested groups deviated from each other, indicating that significant metabolic alterations were introduced by sepsis and treatment of PBA. The metabolic profile in the PBA group deviated from the model group and closed to the control group, indicating that PBA exerted potential protection from metabolic disturbances induced by sepsis.

**Fig. 2:**
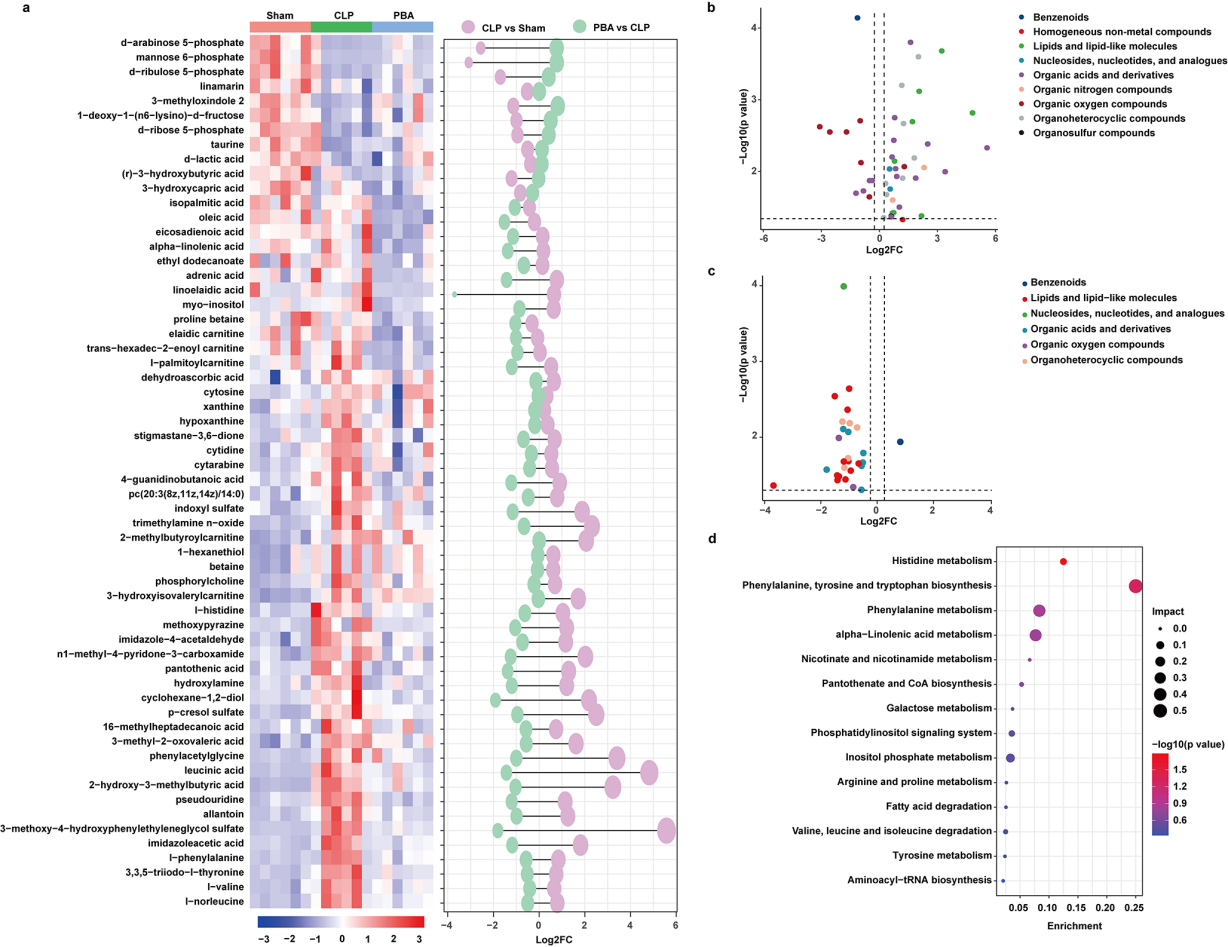
The differential metabolites in septic rats treated by PBA. (**a**) The heat maps and fold change dumbbell charts of potential metabolites. (**b**) Volcano plot of different metabolites between the sepsis and Sham groups. (**c**) Volcano plot of different metabolites between the PBA and sepsis groups. (**d**) The metabolic pathways of significant metabolites

OPLS-DA was used to sharpen the difference between the three groups further (Fig S1d). The corresponding S-plot was applied to observe and screen differential variables, of which the variable importance (VIP) in the projection value was used to evaluate their contribution to the model (Fig S1e and f). Based on the criteria of *p*-values < 0.05 and VIP > 1.0, total 60 metabolites were differentially expressed, including 47 differential metabolites between sepsis and Sham group (36 down-regulated and 11 up-regulated) and 28 differential metabolites between sepsis and Sham group (27 down-regulated and 1 up-regulated) (Fig. 2a). As compared with sepsis, differential metabolites after PBA administration were major involved in lipid and lipid molecules (12 metabolites), Organic acids and derivatives (7 metabolites), and organic heterocyclic molecule (5 metabolites). Among these, the major decreased metabolites were fatty acyls, carboxylic acids and derivatives, while the increased metabolites were phenols (Fig. 2b-c; Table 1). The differential metabolites between the PBA group and the sepsis group were imported into the Metaboanalyst to explore metabolic pathway analysis. The results showed that histidine metabolism, phenylalanine, tyrosine and tryptophan biosynthesis, phenylalanine metabolism, alpha-linolenic acid metabolism and inositol phosphate metabolism were the major pathways enriched (Fig. 2d). These results indicated that PBA protected myocardium in sepsis by regulating amino acid metabolism and lipid metabolism.

**Table 1:** Differential metabolites of PBA/sepsis in heart

Metabolite classification		Metabolite	sepsis/Sham		PBA/sepsis	
Sueprclass	Class		*P*	VIP	*P*	VIP
Benzenoids	Phenols	3-methyloxindole 2	7.22E-05	1.69482	0.011467	1.33377
Lipids and lipid-like molecules	Fatty Acyls	2-hydroxy-3-methylbutyric acid	0.000209	2.6562	0.020648	1.89469
		alpha-linolenic acid	0.630574	0.82302	0.032826	1.95053
		leucinic acid	0.001528	2.53663	0.03188	1.90297
		linoelaidic acid	0.491946	1.27863	0.04335	1.92851
		eicosadienoic acid	0.61589	0.70013	0.036013	1.37068
		elaidic carnitine	0.777954	1.8379	0.002266	4.5546
		ethyl dodecanoate	0.684041	0.69229	0.022169	1.48902
		isopalmitic acid	0.128342	5.93047	0.004311	8.52666
		l-palmitoylcarnitine	0.182274	3.67516	0.02088	5.3655
		oleic acid	0.497573	3.63358	0.00285	7.22357
		trans-hexadec-2-enoyl carnitine	0.913744	1.2173	0.027549	2.14897
		adrenic acid	0.174727	2.79183	0.036824	3.58793
Nucleosides, nucleotides, and analogues	Nucleoside and nucleotide analogues	pseudouridine	9.95E-05	0.98679	0.0001	1.07083
Organic acids and derivatives	Carboxylic acids and derivatives	3,3,5-triiodo-l-thyronine	0.003683	1.52773	0.02154	1.36091
		4-guanidinobutanoic acid	0.011769	1.04805	0.007763	1.24384
		l-norleucine	0.001781	2.12165	0.016096	1.8026
		proline betaine	0.465004	0.92909	0.008476	1.46948
		l-phenylalanine	0.009173	1.67337	0.049383	1.4683
	Keto acids and derivatives	3-methyl-2-oxovaleric acid	0.000158	1.75304	0.023818	1.19185
	Organic sulfuric acids and derivatives	3-Methoxy-4-hydroxyphenylglycol 4-sulfate	0.00469	1.19899	0.026756	1.03702
Organic oxygen compounds	Organooxygen compounds	myo-inositol	0.100937	0.87344	0.045537	1.02908
		pantothenic acid	0.00859	2.94479	0.010173	3.23157
Organoheterocyclic compounds	Azoles	allantoin	0.002151	3.61421	0.006492	3.51453
		imidazole-4-acetaldehyde	0.00063	2.98401	0.007401	2.58098
		imidazoleacetic acid	0.006501	1.37343	0.025238	1.2165
	Diazines	methoxypyrazine	0.012423	1.38981	0.018871	1.44152
	Pyridines and derivatives	n1-methyl-4-pyridone-3-carboxamide	0.000252	2.53852	0.006198	2.28156

### The mechanism of PBA regulating metabolites in sepsis

**Fig. 3:**
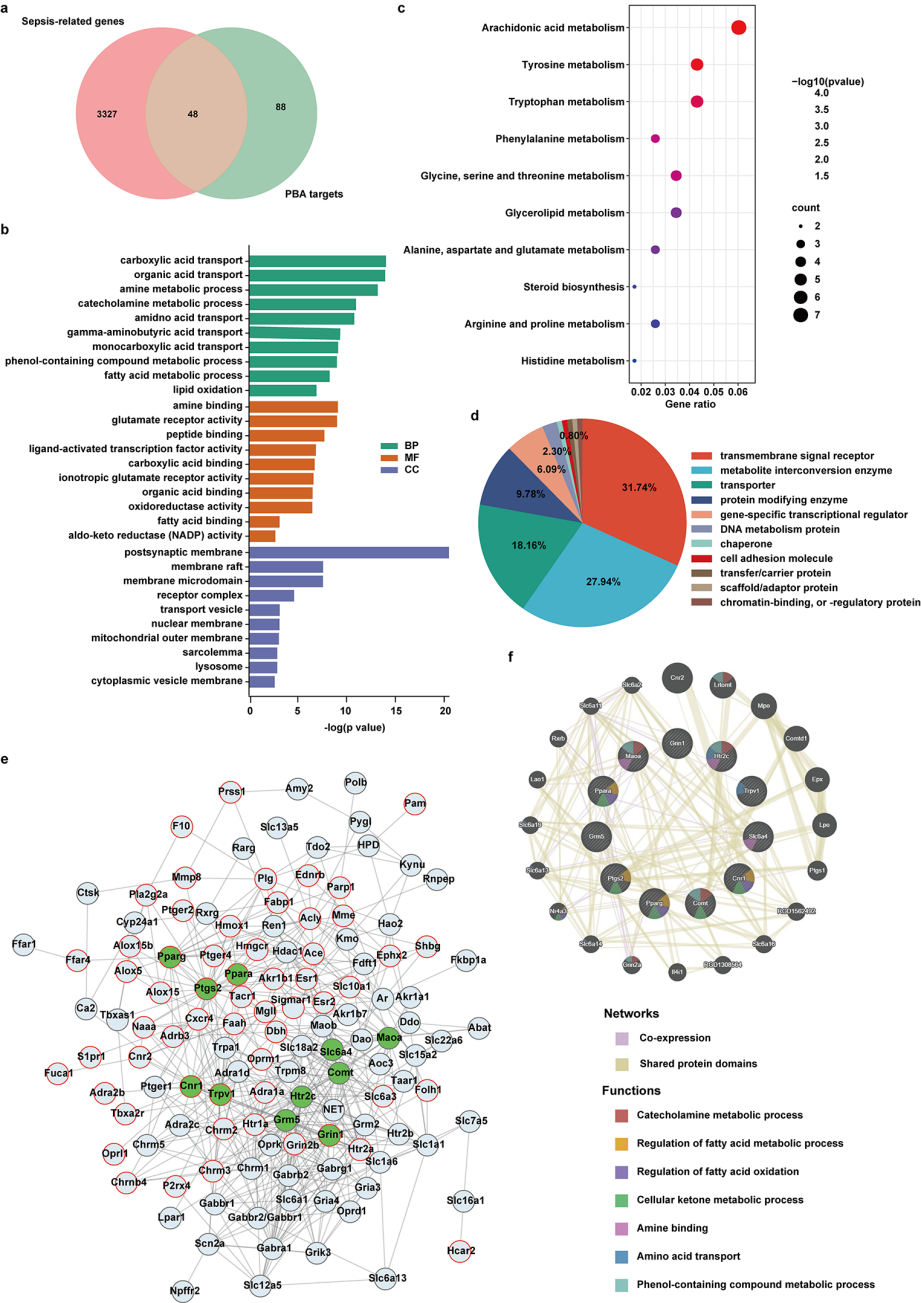
Network pharmacology analysis of PBA treating sepsis. (**a**) Venn diagram of the intersection of sepsis-related gene and PBA-related gene. (**b**) Gene ontology (GO) results of biological process (BP), cellular component (CC), and molecular function (MF). (**c**) Biological processes were enriched by FunRich software **v**.3.1.3. (**d**) The PANTHER Classification System analysis on potential targets genes. (**e**) PPI network of PBA treatment on sepsis. Green, hub genes. Red edges, disease genes. (**f**) Gene–gene interaction networks and functions of 11 hub genes in GeneMANIA

The above results showed that a variety of metabolites altered in septic rats following PBA treatment. To further explore the mechanisms of PBA switching these metabolic patterns, we then searched for potential targets of PBA by network pharmacology. 3375 sepsis-related genes were collected from GeneCards database and DisGeNET database, and 137 potential targets of PBA were obtained from SEA database and Swiss Target database, one-third of PBA’s target genes were disease genes (Fig. 3a). Gene Ontology (GO) term analysis revealed that the enrichment of above genes participated in several metabolic process, including amido acid transport, lipid oxidation and fatty acid metabolism process (Fig. 3b and c). Cellular component analysis showed that these genes were mainly related to transport vesicle, receptor complex, membrane microdomain, and so on. Further molecular function analysis found that the common genes were engaged in amine binding, carboxylic acid binding, organic acid binding and fatty acid binding. The protein classes encoded by these genes were determined using the PANTHER classification system, much of which belonged to metabolite interconversion enzyme, transmembrane signal receptor and protein modifying enzyme (Fig. 3d). These results further testified that PBA exerted its protective role by regulating lipid metabolism and amino metabolism in sepsis.

To gain further insight into PBA regulating myocardial metabolism in sepsis, a PPI network of the 136 targets of PBA was constructed to establish the interaction between these targets using the STRING database. As shown in Fig. 3e, 126 of 136 genes interact with each other to form a PBA targets network (551 nodes), with 59 of them being disease genes associated with sepsis. The centrality measures for nodes in the target network were computed using the Cytohubba App of the Cytoscape software. The centrality of nodes was then ranked applying 3 global parameters based on shortest paths (Closeness, Betweenness, and Degree), 11 hub genes were selected including Grin1, Comt, Slc6a4, Maoa, Htr2c, Grm5, Cnr1, Ppara, Pparg, Ptgs2, Trpv1. To further investigate their networks and functions, GeneMANIA was applied to construct their gene networks. A total of 20 nodes representing genes were associated with the above 11 hub genes in co-expression and shared protein domains. Further functional analysis revealed that these hub genes were significantly correlated with catecholamine metabolic process, regulation of fatty acid metabolic process, regulation of fatty acid oxidation, cellular ketone metabolic process, amine binding, amine acid transport, and phenol-containing compound metabolic process (Fig. 3f). These results suggest that these hub genes play an important metabolic regulatory role in sepsis.

### Key genes of myocardial metabolic alteration following PBA administration

**Fig. 4:**
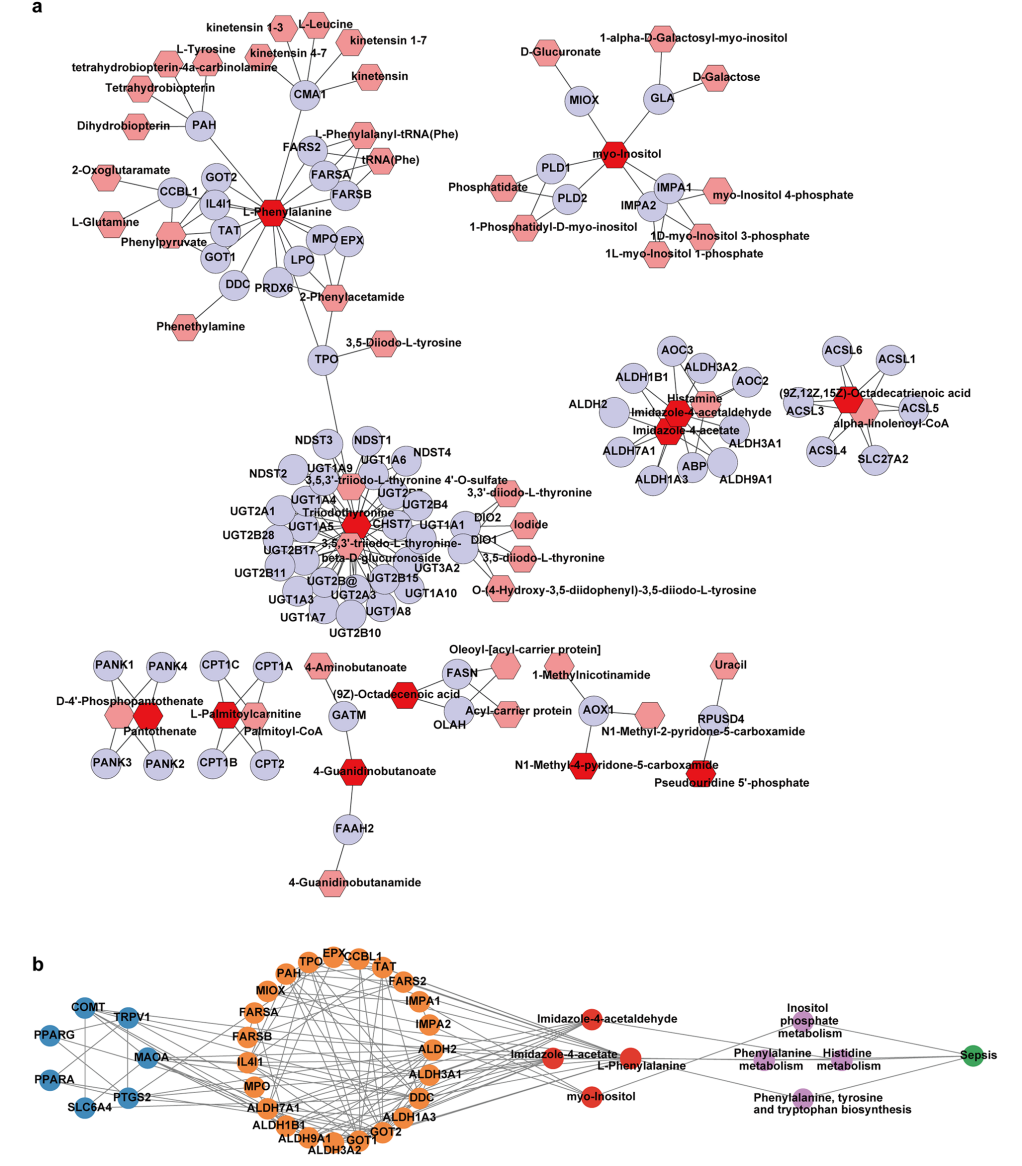
The network of PBA for its anti-sepsis effect. (**a**) Metabolite-gene network of PBA for its anti-sepsis effect. Red nodes, pink nodes, blue nodes, edges represent differential metabolite, interacting metabolite, interacting protein, biochemical reaction, respectively. (**b**) The multi-level network of PBA. The nodes of orange, purple, yellow, green was used to represent candidate targets, metabolite related genes, differential metabolites, metabolic pathways and diseases respectively

To further identify the key genes related to the changes of metabolites in sepsis with PBA treatment, a metabolite-gene network of the differential metabolites was constructed using MetScape plugin for Cytoscape. We obtained the related genes for 11 metabolites (Fig. 4a). Then, a candidate targets-metabolite related gene-metabolites-metabolic pathways-diseases network was constructed. The results found that PBA regulated phenylalanine, tyrosine and tryptophan biosynthesis, histidine metabolism, phenylalanine metabolism and inositol phosphate metabolism regulating 23 metabolic-related genes by 7 key targets (Comt, Slc6a4, Maoa, Ppara, Pparg, Ptgs2, Trpv1), which led to the changes of 4 metabolites (imidazole-4-acetaldehyde, imidazole-4-acetate, L-phenylalanine and myo-Inositol) and 4 metabolic pathways (phenylalanine, tyrosine and tryptophan biosynthesis, histidine metabolism, phenylalanine metabolism and inositol phosphate metabolism) (Fig. 4b).

### PBA improved the immune microenvironment of septic heart by regulating Comt/Ptgs2/Ppara

**Fig. 5:**
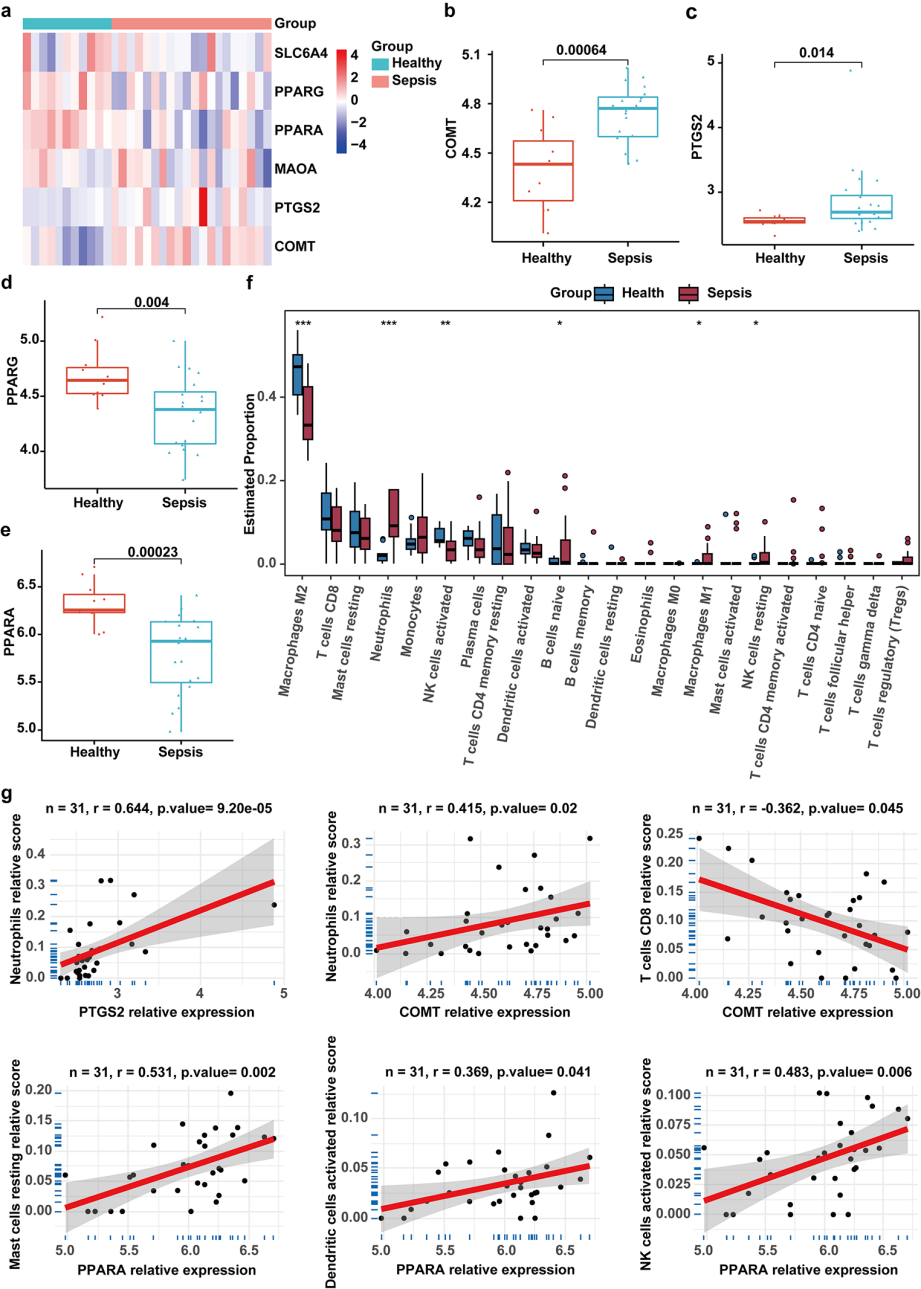
Persistently dysregulated gene expression during sepsis development. (**a**) Heatmap of hub genes in GSE79962. (**b**-**e**) The expression of hub genes in patients with sepsis and normal controls in GSE79962. (**f**) Differences in immune cell infiltration between different groups in the GSE79962 dataset. (**g**) Pearson correlation of immune infiltrating cells with the key genes

The above results revealed that 7 key genes may be involved in the regulation of myocardial metabolism by PBA in sepsis. To further testify the role of these genes, we analyzed RNA-seq datasets GSE79962 from patients with sepsis. We examined the relative expression of the 7 key genes in heart with sepsis (Fig. 5a-e). In patients died from sepsis, the expression levels of COMT and PTGS2 were significantly elevated, PPARA and PPARG were significantly decreased. Meanwhile, the expression of Slc6a4 and Maoa showed no apparent changes. There was no detectable TRPV1 expression in datasets GSE79962. Previous studies demonstrated that immune disorder plays a vital role in the occurrence of sepsis and that metabolic profile determines the immune state. Therefore, based on the expression matrix of dataset GSE79962, we analyzed the effects of the above key genes on immune cell infiltration in patients with sepsis. The results showed that the myocardium of healthy controls and sepsis patients displayed different immune cell infiltration. Compared to the healthy control group, the fractions of M2 Macrophages, activated NK cells had a lower abundance in patients with sepsis, while the fraction of Neutrophils, B naïve cells, M1 macrophages and resting NK cells was significantly higher than those in the control group (Fig. 5f). Next, we analyzed the correlations between the expression of the above hub genes and the abundance of the immune cell types. PTGS2 was significantly positively correlated with neutrophils (*r* = 0.644, *p* = 9.20e-05). COMT was positively correlated with neutrophils (*r* = 0.415, *p* = 0.02) and negatively correlated with CD8 T cells (*r*=-0.362, *p* = 0.045). PPARA showed significant positive associations with Mast cells resting (*r* = 0.531, *p* = 0.002), Dendritic cells (*r* = 0.369, *p* = 0.041) and activated NK cells (*r* = 0.483, *p* = 0.006) (Fig. 5g). These results indicated that PBA could regulate the immune microenvironment of sepsis through the three key genes to participate in the protective effect on myocardium.

**Fig. 6:**
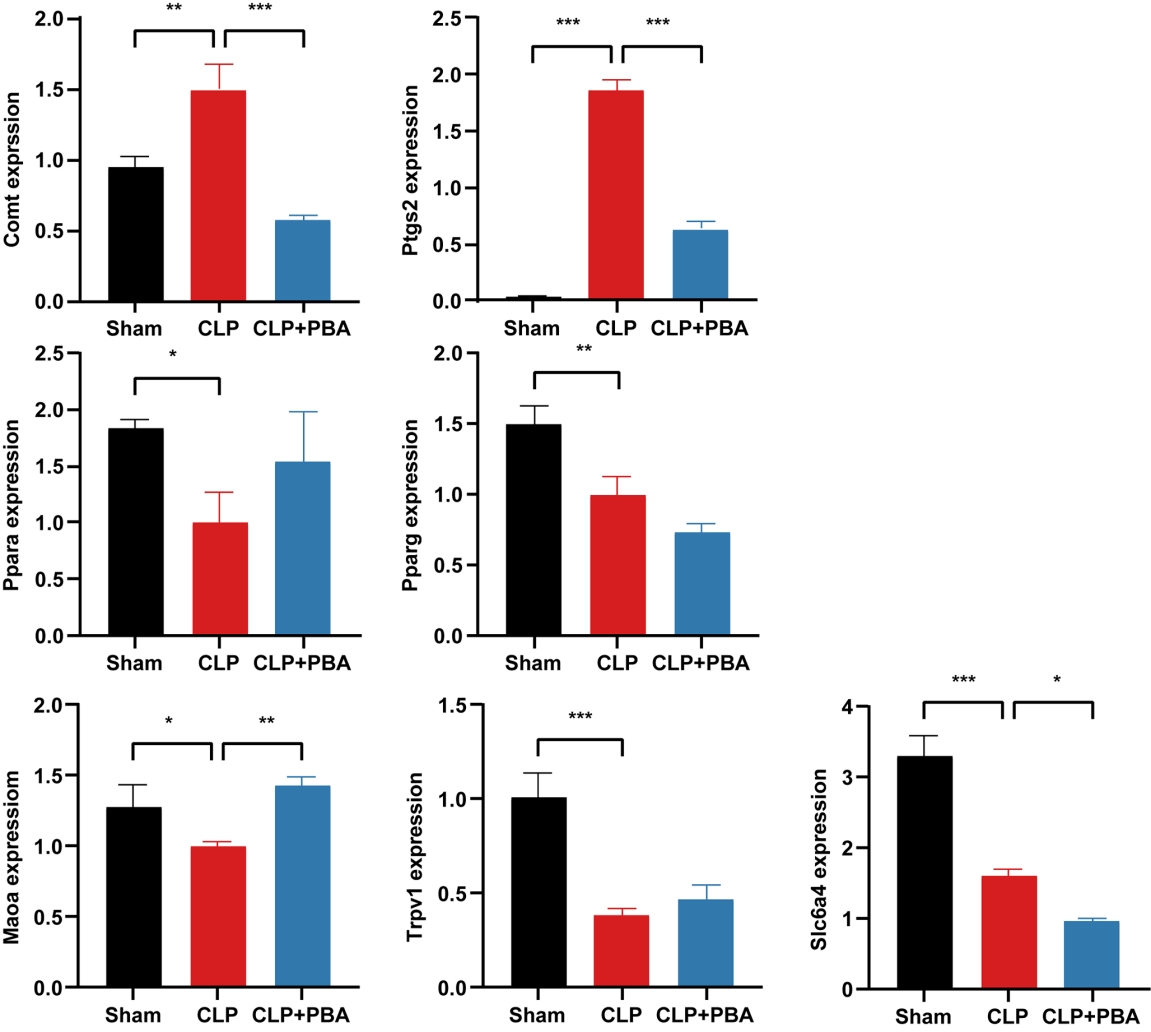
The mRNA expression levels of Comt, Slc6a4, Maoa, Ppara, Pparg, Ptgs2, Trpv1 in rat cardiac tissues. Results are represented as mean ± SD (*n* = 6). *, *p*<0.05; **, *p*<0.01; ***, *p*<0.001

To further verify above findings, we examined the gene expression levels of 7 key genes including Comt, Slc6a4, Maoa, Ppara, Pparg, Ptgs2, Trpv1 in heart from normal, septic and PBA treated rats (Fig. 6). Consistent with the results from the transcriptomics datasets on human cardiac tissues, sepsis increased Comt and Ptgs2 mRNA levels and decreased Ppara and Pparg mRNA levels. Following PBA treatment, the changes of Comt, Ptgs2 and Ppara but not Pparg were largely reversed. Additionally, we found that PBA could rescue the decreased Maoa level in sepsis. PBA also partially restored the decreased TRPV1 level, but no effect on Slca64. These results demonstrated that PBA regulated metabolism-related genes by activating Ppara and inhibiting Comt and Ptgs2, which resulted in the change of amino acid metabolism and lipid metabolism, and then exerted a protective effect on cardiac function in sepsis.

## Discussion

The present study found that PBA improved survival by protecting cardiac function in rats with sepsis and was consistent with previous reports from our laboratory. Metabolism disorder play important role in the sepsis. PBA protected cardiac function by modulating lipid metabolism and amino acid metabolism. 7 key targets (Comt, Slc6a4, Maoa, Ppara, Pparg, Ptgs2, Trpv1), 4 key metabolites (imidazole-4-acetaldehyde, imidazole-4-acetate, L-phenylalanine and myo-Inositol) and 4 related pathways (histidine metabolism, phenylalanine, tyrosine and tryptophan biosynthesis, phenylalanine metabolism, and inositol phosphate metabolism) were showed to participate in PBA protecting cardiac function by combining metabolomics with network pharmacology. The Comt, Ptgs2 and Ppara, were demonstrated as key genes to modulate amino acid metabolism and lipid metabolism to play a protective role on cardiac function in sepsis.

Myocardial dysfunction is a common complication of severe sepsis and septic shock, with an incidence of up to 50%. There may be organic damage to the myocardium at the early of sepsis, which was characterized by ultrastructural changes in cardiomyocytes, impaired myocardial contractile function and cardiac hypertrophy, and was one of the major causes of poor prognosis in patients with sepsis (Carbone et al., 2022). Recent studies suggested that the mechanism of cardiac injury following sepsis involved in myocardial energy metabolism disorders, immunosuppression, cardiomyocyte apoptosis and so on (Zhang & Ning, 2021). Of these, impaired myocardial energy metabolism has been proven to play a very important role in the occurrence and development of cardiac dysfunction following sepsis (Wasyluk et al., 2021). Sugar, lipid and protein are the three major energy substances of the body. In a normal physiological state, fatty acid β-oxidation in the mitochondrion was the preferred energy source in cardiomyocytes. During sepsis, mitochondrial function and structure were damaged, which led the inhibition of key enzymes involving fatty acid uptake, transport, and oxidation, as well as transcription factors modulating fatty acid oxidation. Subsequently, fatty acid metabolism was impaired and mitochondrial oxidative phosphorylation substrates insufficient, further reducing ATP synthesis (Zhu et al., 2022). Furthermore, glucose metabolism provided 20% of cardiac energy under different physiologic state and nutrient conditions. Cardiomyocyte utilized glucose for energy production via aerobic glycolysis in normal condition. During sepsis, GLUT4 was down-regulated and PDK4 was up-regulated in cardiac tissues, which resulted in the inhibition of glucose metabolism, further aggravated the impairment of myocardial energy production and promoted the occurrence and development of cardiac injury in sepsis (Chew et al., 2013). Amino acids did not constitute a high proportion of the energy supply to the normal heart. At present, the relationship between amino acids and sepsis has not been fully clarified. It has been shown that some amino acids such as branched chain amino acids can exist as a coenzyme or coenzyme factor in cardiomyocytes and play a crucial role in the signal transduction of energy metabolism (Li et al., 2020). In ischemic heart failure, there were metabolic abnormalities with an elevated amino acerbity demand (Satomi et al., 2020). This may be the mechanism by which amino acid metabolism affects the prognosis of heart failure.

ER is a vital organelle involved in lipid biosynthesis and protein synthesis, calcium storage, amino acid, and fatty acid metabolism (Addinsall et al., 2018). When fatty acid metabolism disorders, oxidative stress, hypoxia or inflammation attacked the body, there is a mass of unfolded or misfolded proteins accumulated in cells (Guan et al., 2014). This accumulation disrupts homeostasis in the ER and causes ERS. ERS-mediated UPR is closely related to intracellular lipid metabolism and apoptosis signaling pathways (Markouli et al., 2020). Studies have shown that ER stress markers such as GRP78 and CHOP were significantly increased in animal models of sepsis, trauma, severe hemorrhage, and ischemia-reperfusion injury, and their levels were directly related to the degree of organ dysfunction (Li et al., 2022). 4-PBA is a compound with low molecular weight and chaperone activity. Studies have shown that 4-PBA could alleviate LPS-induced inflammatory response by inhibiting NF-kB pathway and Caspase-3 activation (Zeng et al., 2017). Rishi et al. found that 4-PBA appreciably reversed the altered levels of urinary glycoproteins such as THP, OPN, and calnexin in hyperoxaluria rats, restored calcium homeostasis and alleviated ER stress and mitochondrial dysfunction (Bhardwaj et al., 2022). Yanzou Dong et al. found that the expression of CHOP and ATF4 were downregulated by dietary 4-PBA, then attenuating ERS and oxidative stress induced by a HFD or acute ammonia challenge (Dong et al., 2022). Another group found that 27-hydroxycholesterol, a metabolite of cholesterol-induced ER stress and decreased leptin expression which was restored by 4-PBA administration (Marwarha et al., 2012). Moreover, 4-PBA was able to prevent streptozotocin-induced diabetic nephropathy, with decreased hydroxyproline levels (Luo et al., 2010). 4-PBA was also found to have an effect on thyroid metabolism by interacting with the ER resident type 2 deiodinase (Zhang et al., 2019). The present study and our prior findings showed that 4-phenylbutyric acid could protect cardiovascular function and improve survival in septic rats. We further examined the metabolic changes in septic cardiac tissues after PBA treatment. Sepsis caused cardiometabolic dysregulation, including lipids and lipid molecules packing significantly. PBA treatment can significantly improve cardiometabolic dysfunction in septic rats. We have identified 28 differential metabolites between the sepsis and PBA group, which were mainly classified as lipids and lipid-like molecules, organic acids and derivatives and so on. They mainly enriched in amino acid metabolism (phenylalanine metabolism, histidine metabolism, phenylalanine, tyrosine and tryptophan biosynthesis), lipid metabolism (linoleic acid metabolism) and carbohydrate metabolism (phosphatidylinositol metabolism).

Amino acid metabolism plays a crucial role in adaptive and innate immunity, regulating immune cell activation and antibody production (Zhao et al., 2020). It has been suggested that amino acid-induced signaling was dysregulated during bacterial infections (Koh et al., 2015). Patients with sepsis were also accompanied by metabolic disorders and changed amino acids levels in the early stage, which were related to the onset of septic encephalopathy (Deutz et al., 2023). Changes in amino acid catabolism and downstream molecular pathways have become a hot topic in the development of drugs to control inflammation. We observed that intermediates of aromatic amino acid-related pathways, including L-phenylalanine, were significantly elevated in sepsis. Excess L-phenylalanine inhibited protein synthesis, and PBA treatment significantly reduced L-phenylalanine expression.

Metabolomic studies have been limited to listing potential metabolites and related pathways without further exploring the direct relationships between them. To further investigate the mechanism of PBA in the treatment of sepsis, we evaluated the polypharmacological effects of PBA at the molecular level using network pharmacology. We obtained 136 targets for PBA, one half of which were also sepsis-related genes, and their functions were involved in processes such as amino acid transport, lipid oxidation and lipid metabolism. By further validation, we concluded that Comt, Ptgs2 and Ppara were the key genes in the protection of septic heart function by PBA.

Catechol-oxy-methyltransferase (COMT) is a very important metabolic enzyme in the human body, participating in the metabolism of catecholamine neurotransmitters, and playing an equally important role in the metabolism of drugs or xenobiotics while regulating mood and perception processes (Wang et al., 2021). It was found to be closely relate to the development and medication of many types of diseases such as psychiatric and neurological, oncological and cardiovascular diseases, and was an important drug target (Hall et al., 2019). Currently, COMT inhibitors are used in clinical practice in combination with levodopa for the treatment of Parkinson’s disease (Parrales-Macias et al., 2022). In our study, we found that PBA could target Comt and exert cardioprotective effects by inhibiting its expression in sepsis.

Ptgs2, also known as cyclooxygenase-2 (COX-2), acts as a rate-limiting enzyme in the synthesis of prostacyclin PGs and is extensively involved in the physiological processes that maintain dynamic cardiovascular homeostasis (Zhao et al., 2021). COX-2 is an inflammation-inducing mediator with very low activity in normal tissues as well as in cells. Various pro-inflammatory factors, cytokines, and growth factors can induce the high expression of COX-2 (Khan et al., 2022). In the present study, we found that the expression of COX-2 was significantly higher than normal in the heart tissue of septic rats. Consistent with our study, we also found a significant upregulation of COX-2 by analyzing transcriptomic data from cardiac tissue of septic patients. COX-2 is localized at membranes of the ER and the nuclear envelope (Grewal et al., 2005). Enhanced COX-2 expression and production of prostaglandins has been recently associated with ER stress (Yang et al., 2019). A recent study has reported that COX-2 could act as a new partner and modulator of the IRE1α branch of the UPR (Groenendyk et al., 2018). It has been found that heavy metal chromium-induced renal autophagy and injury were closely associated with COX-2 overexpression mediated by endoplasmic reticulum stress eIF2α-ATF4 pathway (Chen et al., 2019). Melatonin selectively inhibited ATF6 and thus COX-2, enhancing endoplasmic reticulum stress-induced apoptosis in human hepatoma cells (Bu et al., 2017). In the present study, we found that COX-2 was one of the excellent potential targets for PBA treatment of sepsis, and PBA treatment significantly reduced COX-2 expression in the hearts of septic rats.

PPARα, the first member of a subfamily of nuclear receptors (PPARs), also includes PPARβ and PPARγ (Seminotti et al., 2023). Upon activation by binding to the ligand, PPAR forms a heterodimer with the retinoid X-like receptor (RXR) to recognize specific DNA sequences and induce expression of target genes (Hellemans et al., 2007). PPARα is involved in a series of physiological processes, including mitochondrial fatty acid oxidation, catabolism, the inflammatory response, and the stress response (Lin et al., 2022). Drosatos et al. demonstrated that LPS activated the JNK signaling pathway in cardiomyocytes and that the JNK-acting substrate cJUN bound directly to the PPARα promoter, inhibiting PPARα expression and thus reducing fatty acid oxidation, leading to impaired energy metabolism in septic myocardium (Drosatos et al., 2016). Standage et al. found that cardiac PPARα was most severely and persistently downregulated in mice during sepsis, and PPARα^−/−^ mice had the most severe cardiac injury at 24 h after LPS injection, indicating that PPARα was critical for cardiac function and survival in mice during sepsis (Standage et al., 2016). Recent studies have identified a possible role of PPARα in the ER stress response. It was shown that activated PPARα exerted a protective effect by inhibiting endoplasmic reticulum stress induced by myocardial ischemia/reperfusion injury (Yuan et al., 2018). In addition, PPARα can inhibit ATF2 transcription to reduce endoplasmic reticulum stress-induced apoptosis in adipocytes, and PPARα plays a key role in endoplasmic reticulum stress functional transition: it can promote cell restoration to homeostasis via PPARα-autophagy pathway in mild endoplasmic reticulum stress, and promote apoptosis via PPARα-CHOP pathway in severe endoplasmic reticulum stress (Xu et al., 2020). Overall, several studies have shown that PPARα activation played a protective role in regulating endoplasmic reticulum stress during disease progression. Consistent with previous reports, we observed that sepsis inhibited PPARα expression, leading to impaired cardiac energy metabolism and impaired cardiac function. We also found that PBA restored PPARα expression, which may further promote cellular restoration of homeostasis through autophagy and improve cardiac function in septic rats.

Given the strong interrelationship between immune dysregulation and altered metabolic processes in sepsis, we also analyzed the relationship between the three key genes and immune cells. We found that the fractions of M2 Macrophages, activated NK cells had a lower abundance in patients with sepsis than in healthy control, while the fraction of Neutrophils, B naïve cells, M1 macrophages and resting NK cells was significantly higher than those in the control group, indicating that acute inflammation and cell immunity play a crucial role in the pathophysiology of sepsis. We further study showed that COMT and PTGS2 were significantly and positively correlated with neutrophils while Ppara was significantly and positively correlated with Mast cells resting. Reprogramming of metabolism has been shown to be associated with the changes of immune cell infiltration proportions. So, we hypothesized that PBA may induce a series of beneficial metabolic changes through the regulation of the immune microenvironment of sepsis by PTGS2, COMT and PPARA. However, how PBA regulates the three genes still needs further study.

## Conclusion

The present study revealed the key targets as well as related metabolites and pathways for PBA to exert therapeutic effects on sepsis. These findings contribute to elucidating the treatment mechanism of PBA on sepsis and provide data and theoretical support for the in-depth study of its mechanism.

### Electronic supplementary material

Below is the link to the electronic supplementary material.

Supplementary Material 1
