# Supplementary material for: 4-phenylbutyric acid improves sepsis-induced cardiac dysfunction by modulating amino acid metabolism and lipid metabolism via Comt/Ptgs2/Ppara
Source: Metabolomics. 2024 Apr 19;20(3):46. doi: 10.1007/s11306-024-02112-3 (PMC11031492; doi:10.1007/s11306-024-02112-3)

Fig S1. Multivariate data analysis from UPLC-MS/MS. (a) PLS-DA scores plot, (b) PLS-DA model validation diagram (Sepsis vs Sham), (c) PLS-DA model validation diagram (PBA vs Sepsis), (d) OPLS-DA scores plot, (e) S-plot of OPLS-DA (Sepsis vs Sham), (f) S-plot of OPLS-DA (PBA vs Sepsis).


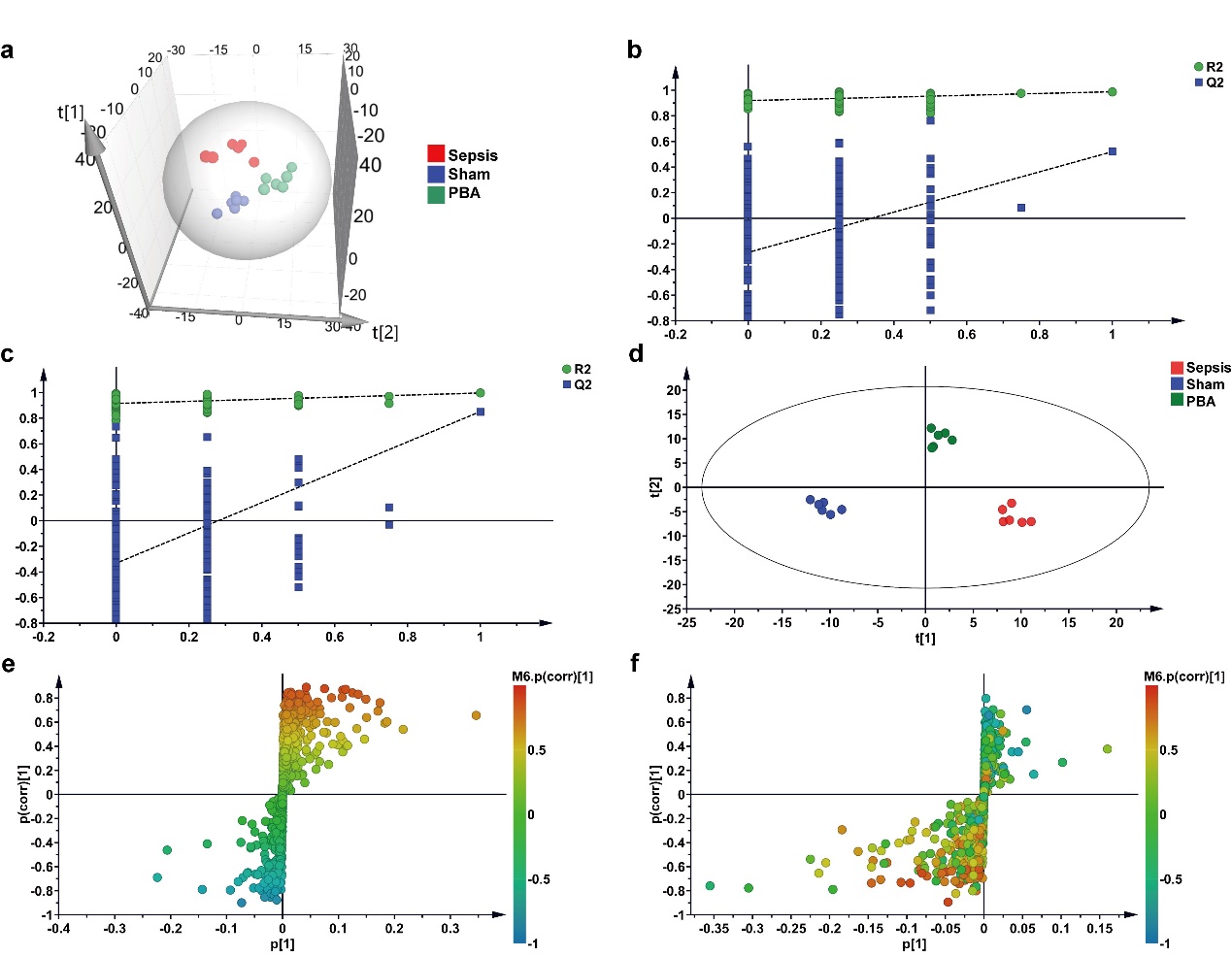

Supplement: Supplementary file 1 — Supplementary Material 1 [file 11306_2024_2112_MOESM1_ESM.docx]
